# LHA-YOLO: A Lightweight and High-Accuracy Detector via Parallel Attention and Divide-and-Conquer Fusion for UAV Images

**DOI:** 10.3390/s26102970

**Published:** 2026-05-08

**Authors:** Jianxiu Yang, Xiong Pan, Qingzhe Pan

**Affiliations:** 1 School of Physics and Electronics, Shanxi Datong University, Datong 037009, China; 2 College of Computer and Information Sciences, Fujian Agriculture and Forestry University, Fuzhou 350002, China; 3 School of Artificial Intelligence, Xidian University, Xi’an 710071, China; panqingzhe@stu.xidian.edu.cn

**Keywords:** small object detection, attention mechanism, feature extraction, multi-scale feature aggregation, unmanned aerial vehicle

## Abstract

Small-object detection in unmanned aerial vehicle (UAV) images poses significant challenges due to limited pixel representation, complex backgrounds, and insufficient feature discriminability. While one-stage detectors like YOLO offer a favorable speed-accuracy trade-off, their performance on small objects is often hampered by conflicts between semantic and spatial information during multi-scale feature fusion in existing networks. To address this, we propose LHA-YOLO, a lightweight and high-accuracy network based on YOLO11. The network is built upon two core innovations. The first is the Lightweight Feature Extraction Module (LFEM), which employs a parallel spatial-channel attention mechanism to extract discriminative cross-dimensional features efficiently and with low computational cost. The second is the Divide-and-Conquer Propagation Path (DCPP) strategy. This strategy decouples and separately optimizes the handling of semantic and spatial information within its bidirectional propagation paths. To achieve this, the top-down path utilizes the Channel Attention-guided Semantic Aggregation (CASA) module to enhance semantic consistency. In parallel, the bottom-up path employs the Spatial Attention-guided Detail Aggregation (SADA) module to preserve spatial fidelity. Extensive evaluation on the VisDrone and UAVDT datasets shows that LHA-YOLO strikes a favorable balance between performance and efficiency. On VisDrone, it improves mAP50 from 39.4% to 41.6% and mAP50–95 from 23.5% to 24.9% over YOLOv11s. On UAVDT, it raises mAP50 from 32.2% to 36.9% and mAP50–95 from 19.4% to 22.9%, while reducing GFLOPs from 21.3 to 18.8. These results confirm the efficacy of our design for real-time UAV applications.

## 1. Introduction

The rapid development of unmanned aerial vehicle (UAV) technology has significantly expanded its applications in fields such as precision agriculture [1], traffic monitoring [2], and disaster response [3]. A key enabler of these UAV applications is real-time object detection, especially small-object detection. Traditional methods for small-object detection in UAV imagery typically rely on handcrafted features [4] and sliding window designs [5], which often suffer from limited generalization capability and insufficient real-time performance. For instance, multi-threshold binarization has been proposed as an effective feature extraction technique, reducing training samples without losing accuracy [6]. In recent years, convolutional neural networks (CNNs) have advanced the field owing to their powerful representational capacity and real-time inference ability. CNN-based methods for small-object detection are primarily categorized into two-stage and one-stage models. Although two-stage detectors [7,8,9] based on Faster R-CNN [10] and Cascade R-CNN [11] generally achieve higher accuracy in detecting small objects, their high computational cost and slow inference speed make them unsuitable for real-time applications. As a result, one-stage detectors [12,13,14] based on YOLO series [15,16] and SSD [17] remain the most widely adopted solutions in practice. These one-stage models are capable of performing detection tasks at high speeds, with YOLO series in particular demonstrating a favorable balance between speed and accuracy. However, detecting small objects in UAV images remains a challenging task due to limited pixel representation of objects, complex backgrounds, and a lack of discriminative features. In response, various attention mechanisms [18,19,20] have been developed to alleviate these issues, as they can adaptively select key information by leveraging the local and global information of the image to improve feature discriminability and assist in feature extraction.

In this paper, we propose a Lightweight Feature Extraction Module (LFEM) that leverages a parallel spatial-channel attention mechanism to effectively correlate cross-dimensional features, thereby capturing discriminative features of small objects. The module is constructed primarily using convolutional blocks, multiple Multi-Dimension Feature Representation (MDFR) blocks, and residual connections to form a residual structure. This design preserves feature map information while sequentially processing it through *n* MDFR groups, facilitating the integration of original features with multi-stage semantic information to provide rich and hierarchical representations. Each MDFR employs a strategy where the input features are split into two equal parts, one for spatial detail extraction and the other for channel-wise semantic processing. Through effective concatenation, features from both dimensions are comprehensively combined at each pixel location, enhancing the representation of small-object-related features and improving computational efficiency. The proposed LFEM effectively captures spatial distributions of objects while simultaneously modeling inter-channel variations, thereby increasing discriminability among object categories.

In order to further enhance the feature representation of small objects, classic object detection algorithms use PA-FPN [21] or Bi-FPN [22] structures to fuse different levels of features. The PA-FPN employs a top-down pathway within the FPN [23] framework to propagate high-level semantic information to lower-level features, thereby enriching their semantic representation. Simultaneously, the PAN module introduces a bottom-up pathway that transmits fine-grained localization and detailed features from lower to higher levels, enhancing the spatial details of high-level feature maps. This design enables effective integration of both semantic and spatial information across different scales, leading to significant improvements in multi-scale object detection performance. However, the classic PA-FPN or Bi-FPN does not fully differentiate the functional differences between top-down and bottom-up information flows when directly merging multi-scale features. This may lead to conflicts between semantic and spatial information, especially impairing the detection accuracy of small objects. Given that the top-down path is mainly used to transmit contextual semantic information, it is necessary to further strengthen the semantic consistency between multi-scale features in this path to fully leverage its capacity for semantic modeling. Similarly, the bottom-up pathway should emphasize the preservation and highlighting of spatial details, particularly localized features essential for precise positioning.

To resolve the conflict between semantic and spatial information, we propose a Divide-and-Conquer Propagation Path (DCPP) strategy that processes the two information streams separately without introducing additional computational overhead. Different from the conventional direct fusion in the classic Bi-FPN structure, our DCPP employs a progressive feature aggregation method and replaces the traditional general attention mechanism with task-specific attention modules in different aggregation processes. Specifically, within the top-down propagation pathway, the Channel Attention-guided Semantic Aggregation (CASA) module utilizes a channel attention mechanism to dynamically recalibrate channel-wise weights. This emphasizes semantically relevant features while suppressing responses from redundant or noisy channels. Subsequently, a progressive aggregation strategy fuses multi-scale features in a stepwise manner, which is essential for maintaining semantic consistency throughout the top-down flow and improving both the efficiency and coherence of contextual semantic propagation. As a result, the CASA module not only enhances semantic alignment across different feature scales but also boosts the discriminative power of the fused representations. Correspondingly, in the bottom-up pathway, the Spatial Attention-guided Detail Aggregation (SADA) module focuses on the spatial dimensions of feature maps using a spatial attention mechanism. This preserves and enhances detailed information, particularly fine-grained features critical for accurate localization. The same progressive aggregation strategy then fuses the refined features, preserving the integrity of spatial details as they propagate upward and thereby improving both the efficiency and fidelity of the bottom-up information flow. By adopting this divided yet complementary bidirectional propagation strategy, our model achieves notable improvements in detection accuracy, especially for small objects against complex backgrounds in UAV images.

With the recent introduction of the YOLO11 model [24], we adopt YOLO11 as our baseline to evaluate its performance in small-object detection scenarios. We integrate the proposed LFEM and DCPP modules into the Backbone and Neck of YOLO11, forming a lightweight and high-accuracy network named LHA-YOLO. This enhanced network improves the capture of discriminative features from small objects while suppressing background noise, thereby strengthening feature extraction and representation capabilities. As a result, LHA-YOLO achieves notable improvements in detecting small objects in UAV images. The main contributions of this work can be summarized as follows:

(1) We propose LHA-YOLO, a YOLO11-based multi-scale feature fusion network that integrates a novel feature extraction module and a aggregation strategy to enhance the accuracy and real-time stability of small-object detection for UAV applications.
(2) We design a Lightweight Feature Extraction Module (LFEM) that employs a parallel spatial-channel attention mechanism to effectively correlate cross-dimensional representations, thereby efficiently extracting discriminative features for small objects with enhanced accuracy and minimal computational overhead.
(3) We present a Divide-and-Conquer Propagation Path (DCPP) strategy, which decouples the processing of semantic and spatial information into dedicated pathways to address their inherent conflict, thus achieving enhanced discrimination and localization for small objects without adding notable computational cost.
(4) We extensively evaluate the proposed method on challenging VisDrone and UAVDT datasets, achieving competitive results in both accuracy and inference speed, demonstrating its practical value for real-time UAV applications.

## 2. Related Work

This section reviews and evaluates existing research results from three aspects: the core evolution of the YOLO series, the improvement of small-object detection based on the YOLO framework, and the integration of attention mechanisms into small-object detection methods.

### 2.1. Evolution of the YOLO Series

The YOLO (You Only Look Once) series has been a cornerstone of real-time object detection research, renowned for its exceptional balance between speed and accuracy. Its evolution represents a continuous refinement of architectural design and training methodologies.

YOLOv1 [25] reframed object detection as a single regression problem. It directly predicted bounding boxes and class probabilities from an entire image. This unified architecture enabled end-to-end training and achieved remarkable inference speeds. However, its spatial constraints, such as limited predictions per grid, caused poor performance on small and densely packed objects. Its localization accuracy was also comparatively coarse. YOLOv2 [26] introduced several key improvements, including anchor boxes, batch normalization, and a higher resolution classifier, boosting recall and precision. YOLOv3 [27] adopted a deeper and more powerful backbone network, Darknet-53, which utilized residual connections. Crucially, it incorporated a multi-scale prediction mechanism inspired by FPN [23], detecting objects at three different scales. This architecture fundamentally enhanced its capability to detect small objects and became the baseline for subsequent research.

YOLOv4 [28] integrated numerous effective techniques such as Mosaic data augmentation, the CSPDarknet53 backbone, the PANet neck, and the CIoU loss, achieving state-of-the-art performance without sacrificing speed. Following this, the development of YOLO became more community-driven. YOLOv5 [29] gained widespread industrial adoption due to its superior engineering and flexible PyTorch framework. Subsequently, YOLOX [30] abandoned the anchor-based paradigm by introducing an anchor-free mechanism and a decoupled head, simplifying the pipeline. Building on these advances, YOLOv6 [31] and YOLOv7 [15] further refined the model through innovations such as structural re-parameterization and trainable bag-of-freebies strategies. YOLOv8 [16] further integrated anchor-free design and new loss functions, forming one of the strongest versions currently available. YOLOv9 [32] introduced Programmable Gradient Information (PGI) and a Generalized Efficient Layer Aggregation Network (GELAN) to mitigate the information bottleneck problem in deep networks. YOLOv10 [33] advanced the field by eliminating the need for Non-Maximum Suppression (NMS) during inference, thereby reducing latency and enabling fully end-to-end object detection.

As the latest iteration in the Ultralytics YOLO series, YOLOv11 [24] demonstrated notable improvements in accuracy, speed, and computational efficiency for real-time detection tasks. Building upon advancements from previous versions, YOLOv11 incorporated optimizations and training methodology, making it highly suitable for a wide range of computer vision applications. Experimental results indicated that YOLOv11 had better performance in small-object detection from UAV imagery. Therefore, we selected YOLOv11 as the baseline for the experiments presented in this paper.

### 2.2. YOLO-Based Improvements for Small-Object Detection

Although the multi-scale design of the YOLO series since YOLOv3 has improved small-object detection, significant room for improvement remains for detecting objects that are small, dense, or blurry. This section mainly summarizes the latest improvement research on small-object detection based on classic architectures such as YOLOv5, YOLOv8, and YOLOv11.

Building upon YOLOv5, SCM-YOLO [34] introduced innovative lightweight modules that enhance spatial local information and adaptively fuse multi-scale feature information. MFFSODNet [35] introduced an additional prediction head that focuses on tiny objects, replaces the large-object head. They designed MSFEM to capture fine-grained information from small objects and BDFPN to achieve efficient multi-scale feature fusion. BiFPN-YOLO [36] incorporated a BiFPN as a replacement for the conventional PANet, enhancing multi-scale feature fusion. The study explored alternative solutions to the Swish function by evaluating its performance against many other activation functions. Wang et al. [37] designed a fine-grained semantic fusion module and TOCM module. These modules enhance the distinction between background and objects, thereby extending the model’s detection boundaries for both.

Building upon YOLOv8, LGA-YOLO [38] employed a MLKM to broaden the receptive field and enhance local features. It also used a DGAM to capture global contextual information and emphasize vehicle features in complex backgrounds. Luo et al. [39] proposed a channel priority attention dynamic snake convolution module to capture fine-grained details, and incorporated a MPDIoU and a DAT to boost detection efficiency while maintaining computational efficiency. LSOD-YOLO [40] introduced a lightweight cross-layer output reconstruction module that strengthens the integration of shallow and deep features. The authors also adopted a lightweight Dysample that preserves fine image details while keeping low computational overhead. Quan et al. [41] used an attention mechanism to capture long-range dependencies between distant pixels, introduced Slideloss to strengthen the learning of challenging samples, and employed ShapeIoU to improve bounding box regression by incorporating shape and scale awareness.

Building upon YOLOv11, PS-YOLO [42] incorporated an efficient FasterBiFFPN neck network. This network replaced the original PAFPN and enables more effective multi-scale feature fusion. PS-YOLO also introduced a NWDLoss, which uses shared convolutions to learn common features across objects of different scales. Li et al. [43] incorporated an efficient channel attention mechanism to enhance feature discriminability, and modified the loss function to increase the model’s sensitivity to uncertain object regions. PC-YOLOs [44] restructured the hierarchical architecture of YOLO11. They incorporated a P2 layer for small-object detection and removed the P5 layer to reduce computational overhead and decrease model complexity. The authors also introduced a coordinate spatial attention mechanism that captures spatial and positional information critical for small objects.

### 2.3. Attention Mechanisms for Small-Object Detection

Attention mechanisms, which emulate the ability of the human visual system to focus on salient regions, offer a powerful approach to enhance relevant features and suppress redundant information. This capability is especially valuable in the context of small-object detection. For channel attention, the Squeeze-and-Excitation (SE) Network [45] is a foundational work that learns to recalibrate channel-wise feature responses by modeling interdependencies between channels. Integrating SE blocks or similar modules [46] into network architectures [47] enables the network to amplify discriminative features for small objects. For spatial attention, the Convolutional Block Attention Module (CBAM) [48] sequentially infers both channel and spatial attention maps. The spatial attention highlights the location of information regions. After applying multi-scale fusion in networks [49], the focus of the network can be guided to spatial positions that may contain small objects, thereby reducing the interference of cluttered backgrounds.

For self-attention and transformers, the core self-attention mechanism [50] of transformer computes interactions between all position pairs in a feature map, enabling it to capture global contextual information directly. Transformer-based detectors [51] and their CNN hybrids [52] use global context to localize small objects, avoiding the long-range dependency loss of local convolutions. Recent works have further tailored transformer architectures specifically for UAV small-object detection. MSAE–DETR [53] introduces a multiscale adaptive enhancement detection transformer with dual-scale attention and frequency-domain modeling. HMF-DEIM [54] designs a lightweight hierarchical backbone with multi-domain feature blending and real-time inference. DRONet [55] builds on RT-DETR and introduces occlusion-aware modules for dense and occluded aerial scenes. MSA-DETR [56] integrates PercepConv and SODAttention modules to enhance multi-scale feature extraction and spatial attention.

Beyond the application of universal attention modules, some studies have designed specialized attention mechanisms tailored for object detection. For example, feature pyramid fusion attention [57] performs attention-guided feature selection and fusion across different hierarchical levels of a feature pyramid network. Context attention [58] is specifically designed to aggregate contextual information from regions surrounding potential objects. Meanwhile, multi-scale attention [59] enables collaborative reinforcement of feature responses from disparate scales, enhancing discriminability across varying object sizes. In summary, attention mechanisms offer an effective technical pathway for small-object detection by enabling feature recalibration and global relational modeling.

## 3. Proposed Method

### 3.1. Overview of YOLO11

**Figure 1:**
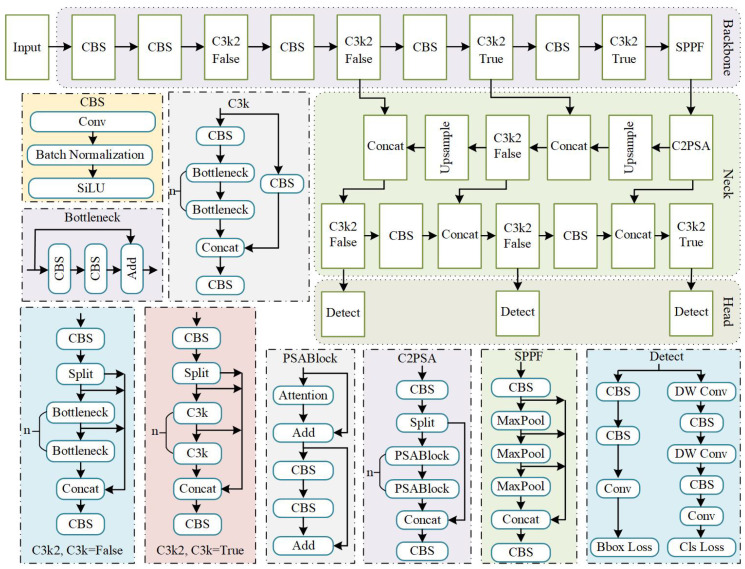
The overall architecture of YOLO11.

Building upon the classic Backbone–Neck–Head architecture, YOLO11 (as shown in Figure 1) introduces significant innovations that achieve steady progress in real-time generic object detection. It achieves an optimal balance between accuracy, inference speed, and computational efficiency through novel network architecture designs and advanced training methods.

The backbone is designed for hierarchical multi-scale feature extraction from input images. Its architecture primarily comprises three key components: CBS blocks, C3K2 modules, and a SPPF module. The CBS block, composed of Convolution, Batch Normalization (BN), and SiLU, serves as a fundamental unit that performs feature transformation and downsampling. Its integrated BN layer and SiLU activation function ensure stable training and expressive feature maps. These features are subsequently processed by the C3K2 modules, which optimize information by splitting feature maps and applying grouped convolutions. The modules can enhance feature representation capacity and computational efficiency. Finally, the SPPF (Spatial Pyramid Pooling Fast) module leverages multiple parallel max-pooling operations with varying kernel sizes to aggregate rich multi-scale contextual information. This effectively improves the model’s ability to recognize objects across different scales without compromising its real-time inference speed.

The neck predominantly employs the classic path aggregation network-feature pyramid network (PAN-FPN) structure, which augments the conventional FPN by incorporating an additional bottom-up path. This pathway effectively reintegrates low-level spatial features with high-level semantic features, thereby enhancing the detection accuracy through multi-dimensional features. Notably, a C2PSA module is introduced prior to feature fusion to leverage attention mechanisms for guided integration. The C2PSA module utilizes multiple parallel attention mechanisms alongside feedforward networks, significantly improving global feature modeling capability. This design enables the network to better capture long-range dependencies and complex nonlinear interactions, ultimately strengthening feature representational power and increasing architectural flexibility across diverse application scenarios.

The detection head is responsible for generating the final predictions, which consist of bounding box coordinates, dimensions, and class probabilities. In the classification branch, depthwise convolution (DWConv) is employed in place of traditional convolution, reducing the number of parameters while preserving accuracy, thereby enhancing the computational efficiency of the model. The regression branch incorporates both standard convolution and deformable convolution to refine the localization performance and improve the accuracy of bounding box predictions. Overall, YOLO11 achieves a superior balance between detection performance and computational efficiency through its refined architectural design and optimized training pipeline.

### 3.2. Overview of the LHA-YOLO Architecture

**Figure 2:**
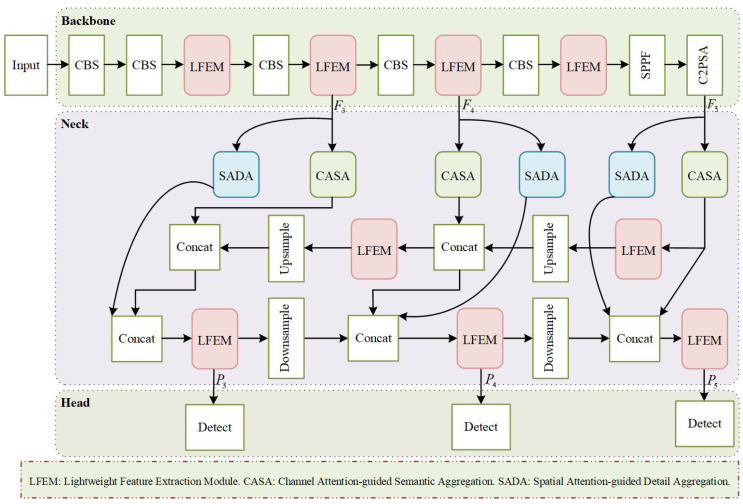
The overall architecture of LHA-YOLO.

Despite its state-of-the-art performance on generic object detection benchmarks, YOLO11 exhibits insufficient feature representation capabilities when applied to small-object detection in UAV images. This paper proposes LHA-YOLO as shown in Figure 2, a novel framework to overcome these shortcomings. The key research objectives include: designing a dedicated attention mechanism to effectively extract and enhance features of small objects; improving the feature pyramid network to achieve more efficient multi-scale feature fusion, facilitating the integration of deep semantic information with shallow spatial details; and incorporating strategies that enhance detection accuracy while maintaining high computational efficiency.

In the backbone network, our architecture retains the five feature extraction stages and the SPPF module from YOLO11. With the exception of the first stage, each feature extraction block primarily consists of CBS and Lightweight Feature Extraction Module (LFEM). The LFEM is composed of a convolutional layer, *n* repeated Multi-Dimension Feature Representation (MDFR) blocks, and a set of residual connections. It is designed to effectively extract and enhance features of small objects while maintaining high computational efficiency.

In the neck network, we introduce a Divide-and-Conquer Propagation Path (DCPP) strategy to enhance the complementary advantages of both information streams without additional computational cost. Specifically, we integrate dedicated aggregation attention mechanisms into both propagation paths. In the top-down pathway, a Channel Attention-guided Semantic Aggregation (CASA) module strengthens semantic consistency across multi-scale features and enhances the discriminative power of the fused representations. In the bottom-up pathway, a Spatial Attention-guided Detail Aggregation (SADA) module progressively refines and aggregates features to emphasize spatial details, particularly the localized features critical for precise localization. This strategy facilitates the effective propagation of both contextual semantics and spatial details, thereby robustly strengthening multi-scale fusion.

As a result, the proposed LHA-YOLO model incorporates specialized modules to achieve a more comprehensive understanding of complex scenes and improve detection performance across diverse object scales.

### 3.3. Proposed LFEM

**Figure 3:**
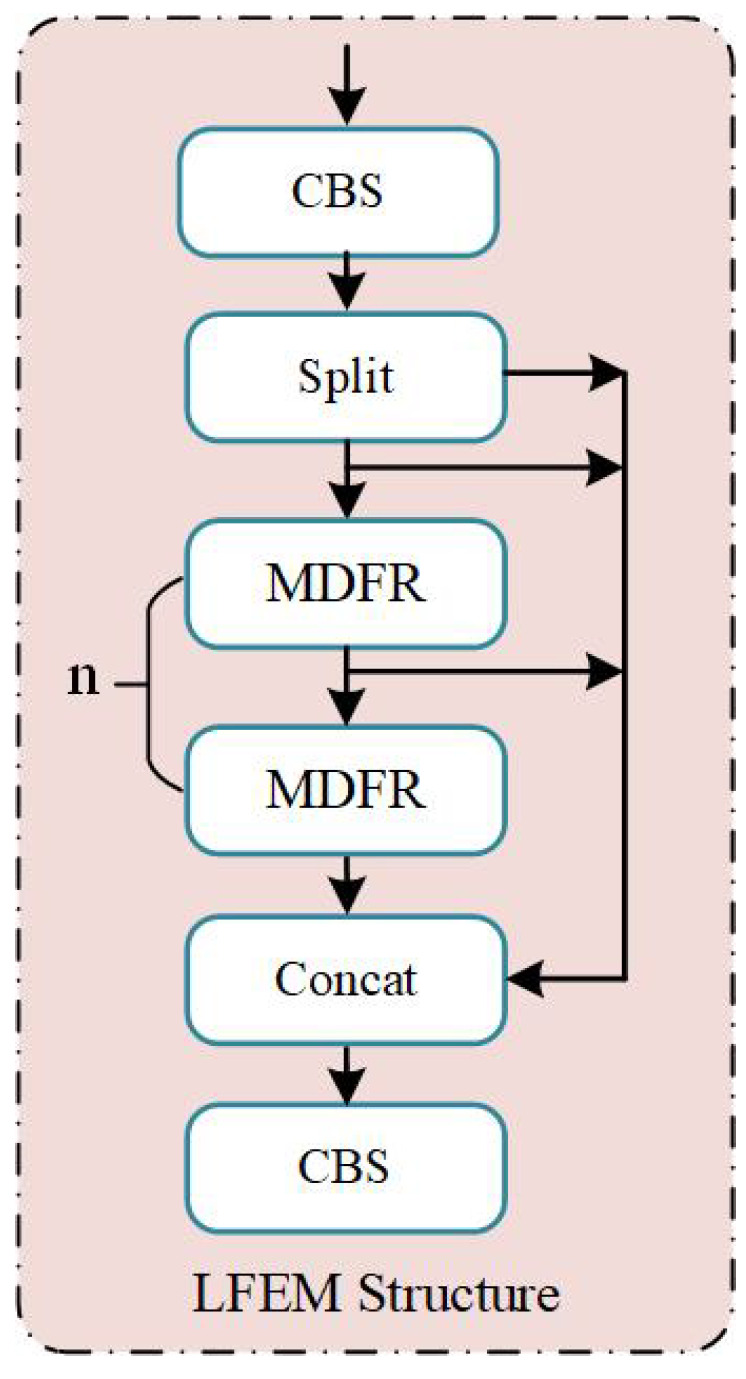
The overall architecture of LFEM.

The Lightweight Feature Extraction Module (LFEM), as shown in Figure 3, is primarily composed of CBS blocks, multiple Multi-Dimension Feature Representation (MDFR) blocks, and residual connections. Within the LFEM architecture, input features are split into two pathways via convolutional operations, one branch is fed into *n* successive MDFR, while the other is retained and later concatenated with the output from these MDFR blocks. As a result, the LFEM enhances multi-channel information integration, thereby improving discriminative feature extraction for small objects.

**Figure 4:**
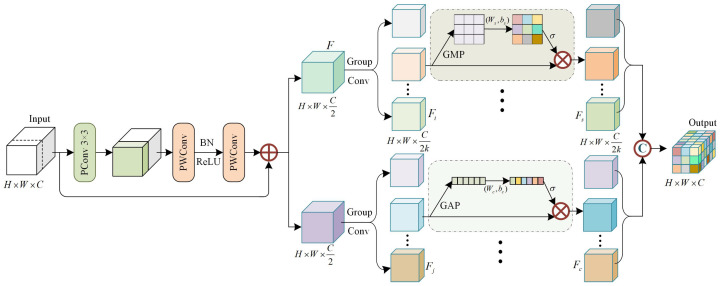
The overall architecture of MDFR.

As shown in Figure 4, the MDFR block consists of two primary stages. The first-stage residual structure begins with a partial convolution (PConv) layer [60] employing a 3×3 kernel, which processes feature maps corresponding to one-fourth of the total channels. These processed channels are then concatenated with the remaining original channels to preserve consistency between the input and output channel dimensions. The combined features are subsequently passed through two pointwise convolution (PWConv) layers to produce the main branch features. Finally, the main branch features are added element-wise to the input feature map to generate the output. This efficient design facilitates comprehensive integration of channel information while maintaining low computational overhead. Specifically, PConv operates efficiently by convolving only a subset of input channels while leaving the remainder unchanged, producing feature maps that combine both original and transformed features. PWConv further compresses channel dimensionality to minimize parameters. Consequently, the LFEM achieves lower computational cost compared to traditional residual structures.

In the second stage, a multi-attention coordination mechanism is employed. The output features from the first stage are split into two separate branches along the channel dimension, each receiving input features *F* of size *H*×*W*×$\frac{C}{2}$, where *H*, *W*, and *C* denote the number of height, width, and channels respectively. Each branch uses multi-scale kernel size to divide the feature map into *k* groups along the channel dimension, denoted as $F_{1}$, $F_{2}$, …, $F_{k}$. The number of channels for $F_{i}$ is $\frac{C}{2k}$. In the upper branch, each subgroup $F_{i}$ is processed by a spatial attention module to capture contextual information for small objects. The attention module learns importance weights that highlight relevant spatial regions. This process is defined as

(1) $$F_{s}=F_{i}\times \sigma (\omega_{s}\cdot GMP\left(F_{i}\right)+b_{s}).$$

$F_{s}$ is the spatial attention feature corresponding to subgroup feature $F_{i}$. σ(·) is the sigmoid activation function. GMP refers to the global maximum pooling operation of $F_{i}$ in the spatial dimension to extract spatial statistics, while $\omega_{s}$ and $b_{s}$ represent a convolutional weight matrix and bias term of size *H*×*W*×1, which aid in encoding spatial representations. Similarly, the lower branch employs a channel attention mechanism to model inter-channel dependencies relevant to small objects. The operation is defined as

(2) $$F_{c}=F_{j}\times \sigma (\omega_{c}\cdot GAP\left(F_{j}\right)+b_{c}).$$

$F_{c}$ is the channel attention feature corresponding to subgroup feature $F_{j}$, which has $\frac{C}{2k}$ channels. GAP denotes the global average pooling operation of $F_{j}$ in the channel dimension, which produces a vector of size $1\times 1\times \frac{C}{2k}$ aggregating channel-wise statistics. The convolutional layer $\omega_{c}$ and bias $b_{c}$ have a kernel size of 1×1 with both input and output channels equal to $\frac{C}{2k}$. The sigmoid output is then broadcasted element-wise to the spatial dimensions of $F_{j}$ for channel recalibration.

Finally, the two branches yield *k* sets of spatial attention and channel attention feature maps, each with dimensions *H*×*W*×$\frac{C}{2k}$. These feature maps are then concatenated and undergo channel shuffling to ensure effective feature redistribution, followed by reconstruction into a new integrated feature representation. Each MDFR incorporates both channel and spatial transformations, enabling effective aggregation of small-object features while suppressing redundant background noise and interference.

### 3.4. Proposed DCPP Strategy

**Figure 5:**
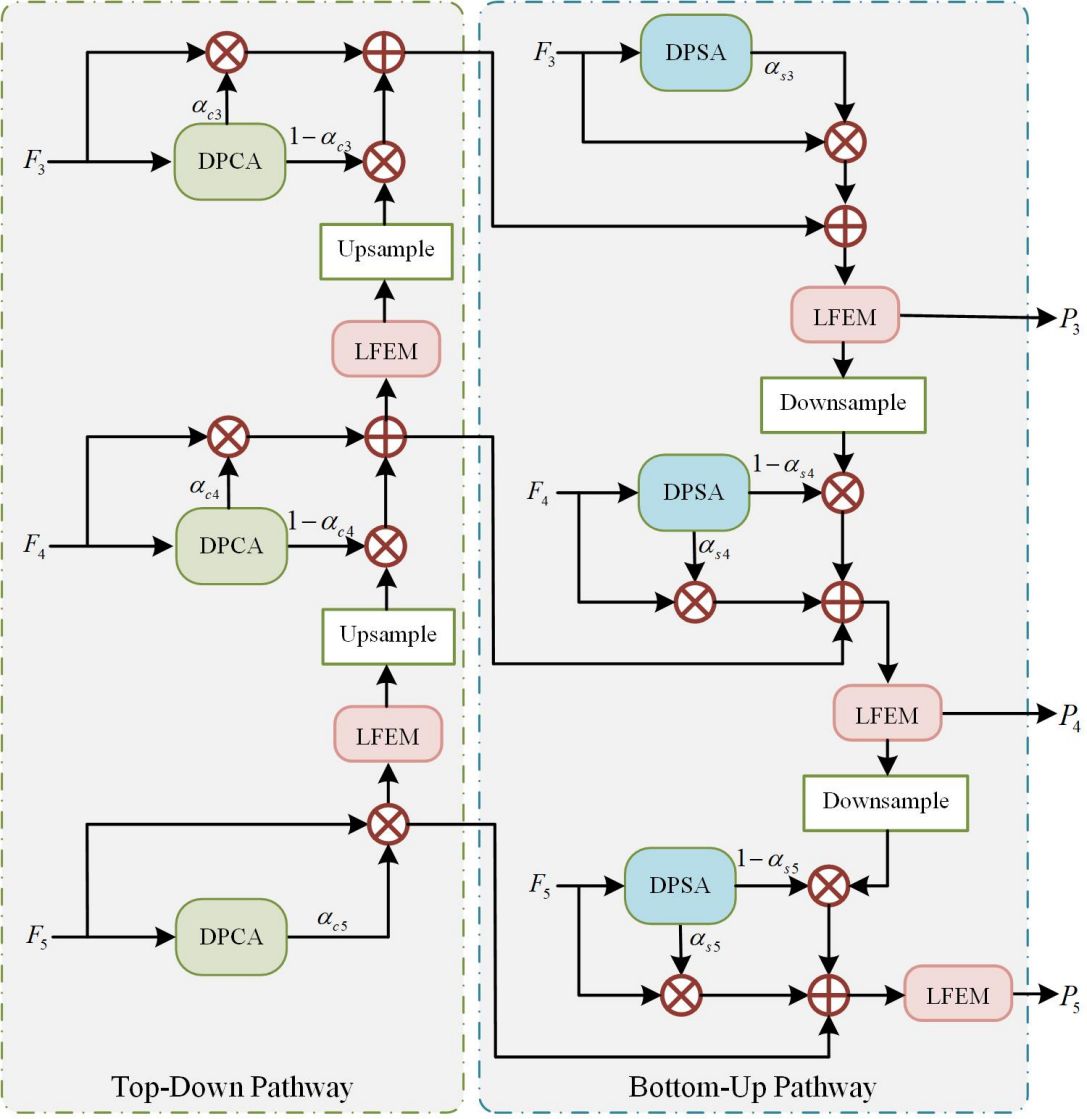
The overall architecture of the DCPP strategy.

As illustrated in Figure 5, the Divide-and-Conquer Propagation Path (DCPP) strategy processes the top-down and bottom-up information streams separately using dedicated attention-guided progressive aggregation. The top-down pathway emphasizes semantic consistency, while the bottom-up pathway preserves spatial details. This divided yet complementary design progressively aggregates contextual semantics and spatial details, thereby significantly enhancing the model’s capacity for multi-scale feature fusion and improving detection accuracy, especially for small objects in complex UAV images.

**Figure 6:**
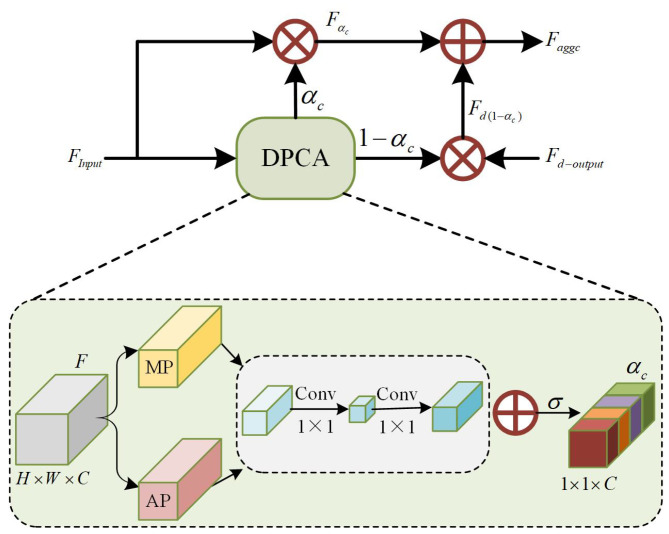
The overall architecture of CASA module. DPCA: Dual-Pooling Channel Attention. MP: Max pooling. AP: Average pooling.

To enhance semantic consistency in the top-down pathway, we propose the Channel Attention-guided Semantic Aggregation (CASA) module, as shown in Figure 6. Its core mechanism dynamically recalibrates channel-wise feature responses to emphasize semantically rich information and suppress noise. Through a stepwise aggregation strategy, the module strengthens cross-scale semantic alignment and boosts the discriminative power of fused features. The Dual-Pooling Channel Attention (DPCA) processes the input features *F* by applying both average pooling and max pooling operations along the spatial dimensions. To minimize computational overhead, the pooled feature maps are first compressed via a shared 1×1 convolutional layer, reducing the channel dimension to $\frac{1}{16}$ of the original. The channel dimension is then restored through another shared 1×1 convolution. The resulting two feature maps are combined via element-wise summation, followed by a sigmoid function to generate the final channel attention weights $\alpha_{c}$. This process is formulated as

(3) $$\alpha_{c}=\sigma \left(\omega_{c}\cdot MP\left(F\right)+\omega_{c}\cdot AP\left(F\right)\right),$$

where MP(·) denotes maximum pooling and AP(·) denotes average pooling. $\omega_{c}$ consists of two 1×1 convolution layers and ReLU activation operations. σ(·) is the sigmoid activation function. The channel-refined features $F_{\alpha_{c}}$ are then obtained by element-wise multiplication of the attention weights $\alpha_{c}$ with the input features $F_{Input}$. Subsequently, the deep-level feature map $F_{d-output}$ is modulated by the complementary weights $\left(1-\alpha_{c}\right)$ to produce a residual feature $F_{d(1-\alpha_{c})}$. Finally, $F_{\alpha_{c}}$ and $F_{d(1-\alpha_{c})}$ are summed to form the aggregated output $F_{aggc}$. This process is formulated as

(4) $$F_{aggc}=\alpha_{c}\times F_{Input}+\left(1-\alpha_{c}\right)\times F_{d-output}.$$

Notably, for the deepest feature map $F_{5}$, the output is generated solely by its channel-wise multiplication with the attention weights. This channel-refined feature is then propagated to shallower layers to convey enhanced semantic information. Consequently, the CASA module refines the quality of semantic propagation throughout the pathway, enriching shallow features with high-level semantics that are pivotal for accurate classification.

**Figure 7:**
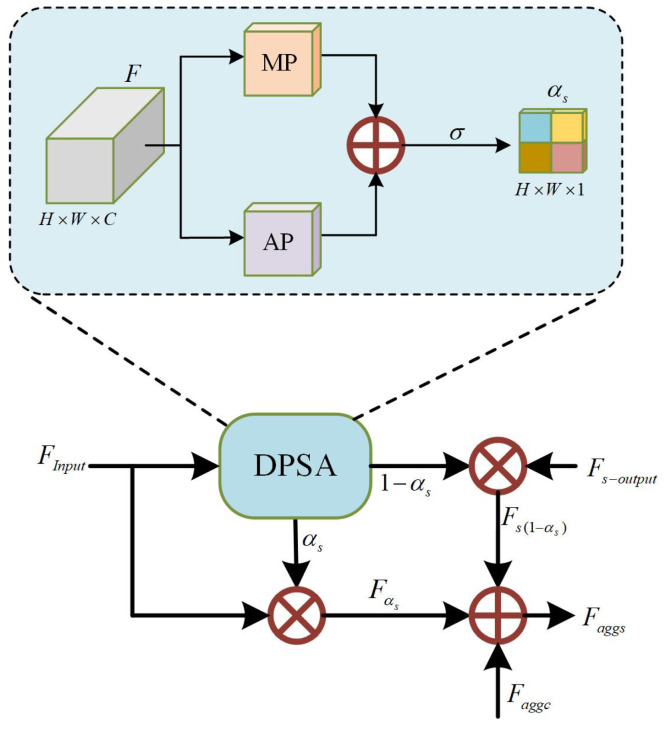
The overall architecture of SADA module. DPSA: Dual-Pooling Spatial Attention. MP: Max pooling. AP: Average pooling.

To preserve and enhance spatial information in the bottom-up pathway, we propose the Spatial Attention-guided Detail Aggregation (SADA) module, as shown in Figure 7. Its core mechanism computes spatial attention weights to model location importance, thereby suppressing background noise while highlighting discriminative features crucial for object localization. Furthermore, a stepwise integration strategy is applied to aggregate these spatial-refined features, which improves both the efficiency and fidelity of spatial detail propagation across the network. The Dual-Pooling Spatial Attention (DPSA) module operates by aggregating channel information from the input features *F* through both maximum and average pooling. To preserve fine-grained spatial details, the input features for this module are sourced directly from the multi-level outputs of the backbone network, consistent with the input to the DPCA module. Then, the pooled maps are fused via element-wise summation, and a sigmoid function is applied to generate the spatial attention weight map $\alpha_{s}$. This process is formulated as

(5) $$\alpha_{s}=\sigma \left(\omega_{s}\cdot MP\left(F\right)+\omega_{s}\cdot AP\left(F\right)\right),$$

where MP(·) denotes maximum pooling and AP(·) denotes average pooling. $\omega_{s}$ denotes the 3×3 convolution and ReLU activation operations. σ(·) is the sigmoid activation function. The input features $F_{Input}$ are spatially refined by the attention map $\alpha_{s}$ to produce $F_{\alpha_{s}}$. Concurrently, the shallow features $F_{s-output}$ are passed through a complementary gate $\left(1-\alpha_{s}\right)$ to form the residual feature $F_{s(1-\alpha_{s})}$, preserving details that the spatial attention may have suppressed. The final aggregated feature $F_{aggs}$ integrates these with the channel-aware feature $F_{aggc}$ via summation, as formulated below:

(6) $$F_{aggs}=\alpha_{s}\times F_{Input}+\left(1-\alpha_{s}\right)\times F_{s-output}+F_{aggc}.$$

For the shallow feature map $F_{3}$, the output is generated solely by its element-wise multiplication with the spatial attention weights. This spatially refined feature is then propagated to deeper layers, conveying enhanced spatial information. By integrating such multi-scale spatial cues that benefit precise localization, the SADA module sharpens the perception of spatial structures in deep features, thereby significantly strengthening the representation and transmission of spatial details throughout the bottom-up pathway.

Unlike standard fusion strategies, our stepwise aggregation performs a weighted combination guided by attention mechanisms, adaptively emphasizing the most informative multi-level features with negligible computational overhead. This approach enhances the bidirectional propagation path, enabling refined feature integration within the pyramid network. Consequently, the model achieves superior multi-scale representation, improving its ability to interpret complex scenes and detect objects across various scales.

## 4. Experimental Results and Analysis

### 4.1. Experimental Datasets

We evaluate our method on two challenging UAV vehicle detection benchmarks: VisDrone [61] and UAVDT [62]. The VisDrone dataset is a standard and challenging small-object detection benchmark designed for advancing research in UAV applications. The dataset encompasses a variety of challenging scenarios, such as crowded city streets, busy traffic intersections, and highly dense pedestrian areas, while also considering various challenging environmental factors, such as nighttime, rainy weather, and foggy weather. This complexity makes it a robust testbed for evaluating detection models. The dataset consists of 8629 images with 6471 for training, 548 for validation, and 1610 for testing, which includes 10 predefined categories such as cars, pedestrians, and bicycles. A notable characteristic of VisDrone is its pronounced class imbalance: cars account for 40.9% of instances, while awning tricycles represent only 0.9%. Importantly, small objects dominate the dataset, comprising 62.4% of all instances. This combination of severe class imbalance and the prevalence of small objects makes accurate detection particularly challenging, aligning the benchmark closely with real UAV-based perception difficulties. UAVDT comprises 39,850 images (23,258 training, 16,592 testing) with three vehicle categories (car, truck, bus) at 1080×540 resolution. Objects are also divided by size into small (area $<32^{2}$), medium ($32^{2}<$ area $<96^{2}$), and large (area $>96^{2}$). The dataset presents significant challenges due to low resolution, illumination variations, partial occlusions, and frequent viewpoint changes caused by UAV motion. These factors, together with the dominance of small and medium sized objects, make accurate detection highly demanding.

### 4.2. Implementation Details

The experimental platform is based on Ubuntu 20.04 operating system, using Python 3.11, PyTorch 2.8, and CUDA 12.8. To ensure fair comparison, all experimental models involved in this paper were trained using the same experimental parameters and settings. All training processes are conducted from scratch without pre-trained weights. The hardware configuration includes i9-11900K CPU and NVIDIA RTX GPU. All models were trained using stochastic gradient descent (SGD) optimizer on the Visdrone dataset for 300 epochs. The parameters of SGD are an initial learning rate of 0.01, weight decay of 0.0005, momentum of 0.937, batch size of 16, and input image size of 640×640.

### 4.3. Evaluation Metrics

Based on standard object detection evaluation metrics, the performance of the proposed LHA-YOLO model and all baseline models was rigorously evaluated on the Visdrone validation set. The main indicators include performance metrics such as precision (P), recall (R), mean average precision (mAP), and complexity metrics such as Million parameters (M), Gigabit Floating Point Operations (GFLOPs), and Frames Per Second (FPS).

Precision measures the accuracy of positive sample predictions. It quantifies the proportion of predicted bounding boxes that correctly classify and locate objects of interest. Recall measures the completeness of model predictions. It quantifies the proportion of actual ground truth objects that are successfully detected by the model. The mean average precision (mAP) is the predominant benchmark metric for evaluating object detection models. It summarizes the accuracy across all object classes by calculating the mean of the average precision (AP) scores for each class. AP is defined as the area under the precision–recall curve for a single category. mAP is the average of AP over all categories. In this paper, we adopt mAP50 and mAP50–95 as the primary evaluation metrics. mAP50 refers to the mean average precision at an IoU threshold of 0.5, while mAP50–95 denotes the average of the 10 IoU thresholds from 0.5 to 0.95 with a step size of 0.05.

### 4.4. Experimental Comparison with YOLO11 Series Models

We comprehensively evaluate LHA-YOLO against YOLO11. As shown in Table 1, all LHA-YOLO variants consistently outperform their baseline counterparts in detection accuracy (P, R, mAP) across different scales. These improvements come with lower computational cost (Params and GFLOPs) and higher inference speed (FPS), especially for the LHA-YOLOl and LHA-YOLOx models.

Taking LHA-YOLOs and YOLO11s as an example, we further analyze per-class performance on VisDrone to assess the impact of class imbalance. Our method improves AP for the majority class, cars, from 79.5% to 81.1%, and for the minority class, awning-tricycles, from 14.7% to 16.5%. These consistent gains confirm that the proposed approach does not sacrifice minority-class accuracy for overall improvement.

**Figure 8:**
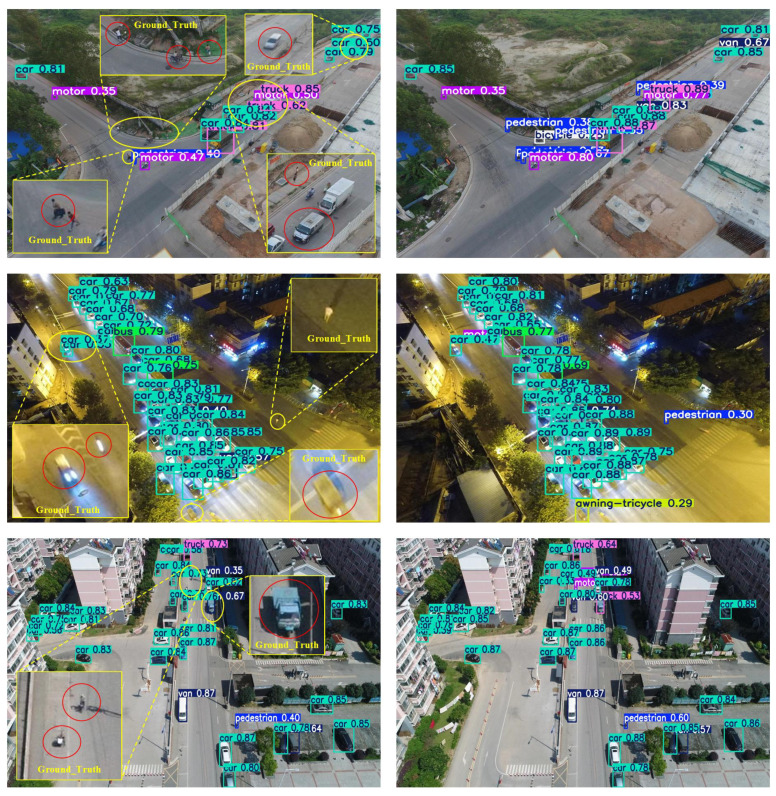
Qualitative comparison between YOLO11s (**left**) and LHA-YOLOs (**right**). The proposed method shows stronger robustness, with fewer false negatives on small objects and suppressed false positives in complex backgrounds.

The superior detection performance of our method is further illustrated through visual comparisons. Figure 8 presents example results between YOLO11s and LHA-YOLOs on challenging scenes. The visualizations reveal that YOLO11s suffers from significant false negatives, failing to detect many small objects. In contrast, LHA-YOLOs successfully identifies these challenging instances with more precise bounding boxes. YOLO11s is prone to generating duplicate or inaccurate detections in complex backgrounds, whereas LHA-YOLOs effectively suppresses such false positives while maintaining a high recall rate. This demonstrates its enhanced capability to discern objects in cluttered environments. These observations strongly support the quantitative metrics, collectively verifying that our proposed improvements substantially enhance detection reliability across diverse UAV scenarios.

### 4.5. Ablation Study

#### 4.5.1. Investigation on Generalizability of LFEM and DCPP Strategy

We conduct ablation studies to validate the generalizability of the proposed Lightweight Feature Extraction Module (LFEM) and Divide-and-Conquer Propagation Path (DCPP) strategy. Both modules are integrated into two distinct detector architectures, YOLO8s and YOLO11s, and evaluated through benchmark experiments under standard protocols.

As shown in Table 2, the proposed LFEM and DCPP strategy bring consistent improvements across both YOLO8s and YOLO11s architectures, with particularly notable gains in the YOLO11s. Notably, their combined use produces a synergistic effect, yielding performance superior to their individual contributions. Specifically, LHA-YOLOs achieves gains of 2.2% in mAP50 and 1.4% in mAP50–95. This demonstrates the compatibility and combined effect of the modules, confirming the generalizability of the proposed approach.

Furthermore, as shown in Table 2, the proposed modules enhance model efficiency. Specifically, integrating the LFEM and DCPP strategy reduces both Params and GFLOPs for both YOLO8s and YOLO11s. The efficiency gains are positively correlated with model size, meaning larger models achieve greater complexity reduction. This effect is clearly observed in the results for YOLO8s. Overall, the findings indicate that our method alleviates the performance–complexity trade-off through structural re-parameterization and computational optimization.

**Figure 9:**
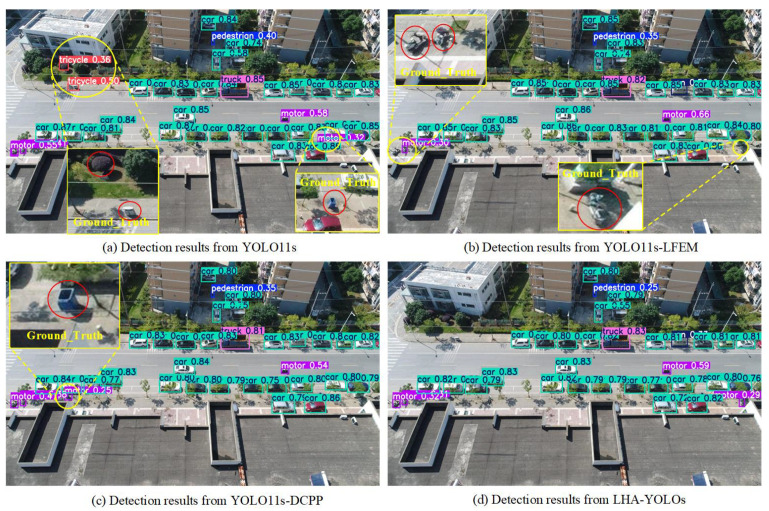
Comparative visualization of module contributions on ground roads. Results from (**a**) baseline YOLO11s, (**b**) YOLO11s-LFEM, (**c**) YOLO11s-DCPP, and (**d**) the integrated LHA-YOLOs.

**Figure 10:**
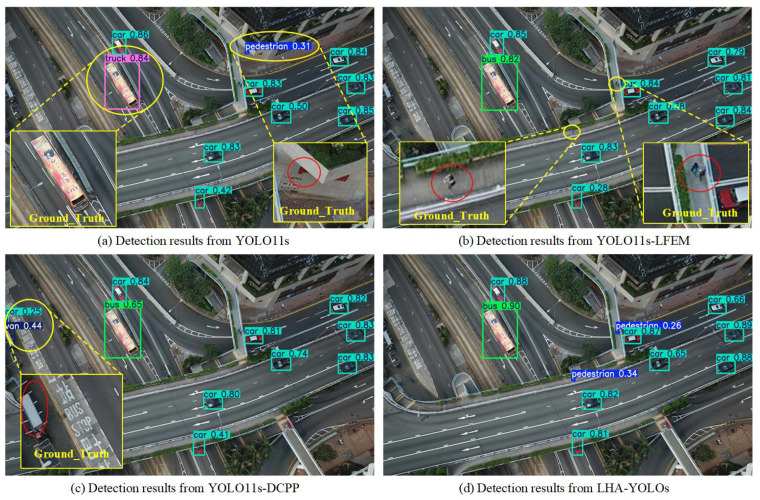
Comparative visualization of module contributions on elevated roads. Results from (**a**) baseline YOLO11s, (**b**) YOLO11s-LFEM, (**c**) YOLO11s-DCPP, and (**d**) the integrated LHA-YOLOs.

We visualize the contributions of each module in different scenarios, specifically on ground roads and elevated roads, as shown in Figure 9 and Figure 10, respectively. Compared to the YOLO11s baseline, the YOLO11s-LFEM model primarily reduces false positives by enhancing feature discriminability but tends to miss some small objects. Conversely, the YOLO11s-DCPP model improves the recall of small objects through better multi-scale feature integration, yet it is prone to generating false detections. The integration of both the LFEM and DCPP strategies in LHA-YOLO yields synergistic benefits, demonstrating that their complementary designs jointly address the core challenges of UAV-based detection.

In summary, the integrated design contributes to cross-architecture generalization. It achieves consistent performance gains alongside complexity reduction in both YOLO8s and YOLO11s. Ablation results indicate that the feature enhancement and attention mechanisms produce complementary effects, which together improve detection accuracy and lower computational costs across different architectural designs.

#### 4.5.2. Analysis of MDFR Effectiveness

This section presents a systematic ablation analysis of the Multi-Dimension Feature Representation (MDFR) block within the LFEM. Experiments are conducted on YOLO11 under identical training conditions, with results detailed in Table 3.

First, the baseline configuration with only the first-stage PConv-PWConv operations provides only modest gains. It exceeds the standard YOLO11m solely in the medium scale, indicating that a design focused purely on efficiency lacks sufficient representational capacity, particularly for smaller models. Subsequently, introducing the channel attention (CA) mechanism brings stable improvements across all scales. CA works by selectively amplifying informative feature channels. Independently, integrating the spatial attention (SA) mechanism also advances the baseline, as SA refines localization by highlighting salient regions and filtering background clutter, which is essential for complex UAV scenes. Ultimately, the complete MDFR block, unifying both attention pathways, delivers the best performance. The parallel CA and SA operations complement each other, in which CA enhances semantic clarity for accurate classification while SA sharpens spatial fidelity for precise regression. This synergy, grounded in an efficient first stage, enables a powerful and manageable multi-scale feature representation.

In summary, the ablation study validates that MDFR’s two-stage design, which combines efficient transformation with dual-attention refinement, achieves a good balance between efficiency and capacity. The progressive performance gain supports the architectural rationale and confirms MDFR’s suitability for real-time, high-accuracy UAV detection tasks.

#### 4.5.3. Evaluation of CASA and SADA Modules Importance

This section aims to evaluate the importance of the proposed Channel Attention-guided Semantic Aggregation (CASA) and Spatial Attention-guided Detail Aggregation (SADA) modules within the DCPP strategy. Experiments are conducted on YOLO11s to assess various attention and aggregation strategies across its bidirectional pathways (Table 4). We test four core components per pathway: Dual-Pooling Channel Attention (DPCA), Dual-Pooling Spatial Attention (DPSA), conventional multi-scale feature fusion (CMFF), and our progressive multi-scale feature aggregation (PMFA). The complete DCPP strategy is realized by coupling DPCA with PMFA in the top-down pathway (CASA) and DPSA with PMFA in the bottom-up pathway (SADA).

The baseline YOLO11s model (row 3) establishes the performance reference. Introducing DPCA in the top-down pathway (row 4) enhances mAP50 by 0.3% and mAP50–95 by 0.2%. Extending this with DPSA in the bottom-up pathway (row 5) yields further gains of 0.5% in mAP50 and 0.5% in mAP50–95. This confirms that integrating attention mechanisms in both pathways enhances detection through complementary feature enhancement, where channel attention emphasizes semantically rich channels and spatial attention amplifies spatially significant regions. However, these CMFF-based improvements remain modest. In contrast, the complete DPCA and DPSA implementation with PMFA (row 6) achieves significant gains, including a 1.0% increase in mAP50, a 0.8% increase in mAP50–95. This demonstrates that PMFA effectively enables DPCA to strengthen semantic coherence and DPSA to preserve structural details.

To validate our pathway-specific design, we tested an alternative that merges DPCA and DPSA outputs via element-wise multiplication. Although this variant surpasses the baseline (final row, Table 4), it underperforms our DCPP strategy. This outcome highlights that the key lies in specialized, pathway-optimized application rather than simple fusion. Our dedicated approach allows each attention type to optimize its designated pathway, where channel attention enhances semantic coherence across scales and spatial attention preserves precise structural details.

In summary, the ablation study substantiates the individual importance of both the specialized attention modules and the progressive aggregation strategy. Theoretically, the conflict between semantic and spatial information arises from the distinct roles of the two pathways. The top-down pathway conveys high-level categorical semantics for classification. The bottom-up pathway captures low-level positional details for localization. Forcing a single fusion node to accommodate both types of information leads to gradient competition and feature interference during training. This is harmful for small objects, which require precise localization. DCPP resolves this conflict by decoupling the two streams. It applies task-specific attention and progressive aggregation to each pathway. As a result, the DCPP strategy integrates CASA and SADA to achieve superior detection performance through an optimal balance of semantic and spatial refinements. This validates the effectiveness of our dedicated architectural design over generic attention combination schemes.

### 4.6. Experimental Comparison with Mainstream Models

We conduct comprehensive benchmarks on the VisDrone and UAVDT datasets to evaluate the generalization of LHA-YOLO against contemporary one-stage, two-stage, and transformer-based detectors.

#### 4.6.1. Evaluation on VisDrone Dataset

Results are summarized in Table 5. Compared to classical two-stage detectors such as Faster R-CNN and Cascade R-CNN, LHA-YOLOs achieves a higher mAP50, with improvements of 22% and 22.7% respectively, while using approximately five times fewer parameters. In the comparison among YOLO series models, we primarily focus on small-scale models. Among them, LHA-YOLOs achieves the best performance on both mAP50 and mAP50–95. While it does not have the lowest Params, its GFLOPs rank second, being only slightly higher than those of FFCA-YOLOs, yet the model still maintains highly competitive overall efficiency. Notably, LHA-YOLOs even outperforms the transformer-based detector RT-DETR-R18, whose parameter count is comparable to the medium-scale model LHA-YOLOm, while LHA-YOLOs uses only half the parameters of RT-DETR-R18. This demonstrates that LHA-YOLO11s achieves an excellent balance between accuracy and model complexity, making it a highly efficient and practical choice for real-world deployment when both performance and resource consumption are considered.

**Figure 11:**
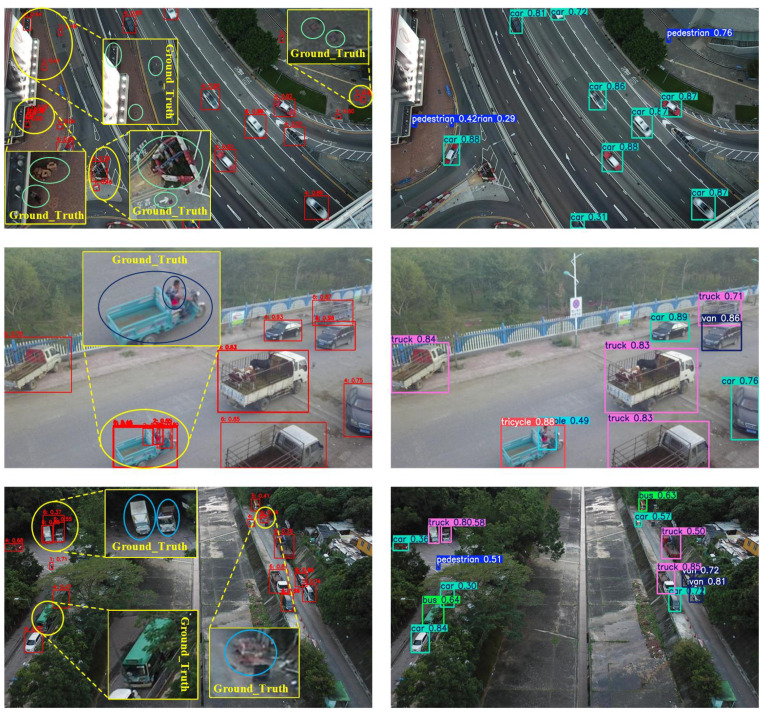
Qualitative detection results comparing LHA-YOLOs (**right column**) and RT-DETR-R18 (**left column**) on VisDrone images.

Figure 11 provides visual evidence of the strengths of LHA-YOLO. In challenging UAV scenarios, it outperforms models such as RT-DETR-R18 by reducing false positives in clutter more reliably and detecting frequently missed small objects more accurately, showcasing its superior robustness. These results verify the reliable performance of our method under real-world complexity, consistent with the quantitative gains.

#### 4.6.2. Evaluation on UAVDT Dataset

**Figure 12:**
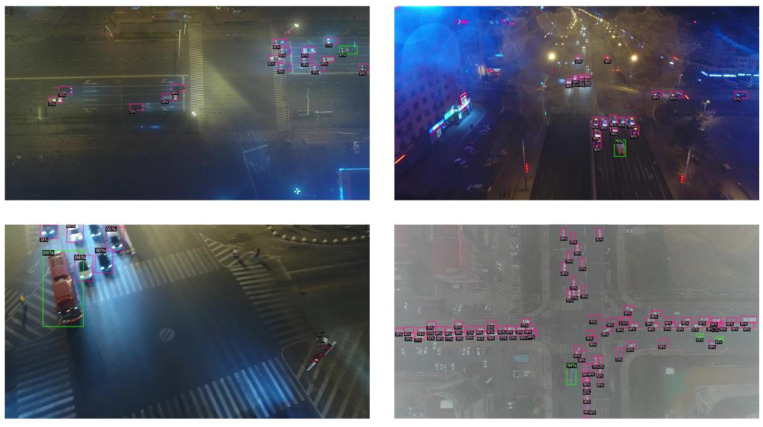
Qualitative detection results on UAVDT images.

To further validate the generalization of our proposed method, we conducted extended experiments on the UAVDT dataset. Table 6 shows the quantitative results. LHA-YOLOs achieves the highest mAP50 36.9% and mAP50–95 22.9% among YOLO series models. Its GFLOPs 18.8 stay competitive among lightweight YOLO variants. Figure 12 provides qualitative detection examples. LHA-YOLOs reliably detects small and dense objects under different lighting and weather conditions. It also handles background clutter well. These results confirm that our method generalizes across different UAV datasets.

### 4.7. Discussion

Based on experimental results, we analyze the performance of LHA-YOLO for UAV object detection. Compared to the baseline YOLO11 models, LHA-YOLO variants consistently improve detection accuracy across all scales. The results are reported in Table 1. The LFEM module reduces computational cost while increasing both mAP50 and mAP50–95. However, the ablation study in Table 3 shows a different finding. Removing the parallel-attention second stage from the MDFR block and keeping only the PConv-PWConv first stage causes a slight mAP drop on YOLO11n and YOLO11s. This indicates that the second-stage attention is essential for recovering representational capacity, especially for small models. The DCPP strategy decouples semantic and spatial information flows using CASA and SADA. This leads to further gains in mAP50 and mAP50–95. Applying DCPP alone without LFEM marginally increases the parameter count. This suggests that the benefit of decoupled propagation is best realized when combined with a lightweight backbone. Together, both modules reduce model complexity. The reduction in GFLOPs is more pronounced than the reduction in parameters. The reason is that DCPP introduces progressive aggregation and additional attention operations. These operations add some parameter overhead but still lower the overall computational cost. In comparisons with mainstream methods on VisDrone and UAVDT, LHA-YOLOs achieves the highest mAP50 and mAP50–95.

Nevertheless, several limitations remain. First, our validation is primarily based on two UAV datasets, VisDrone and UAVDT. Although these are widely used aerial detection benchmarks, the generalization to other small-object scenarios, such as the DOTA dataset for rotating objects, or general surveillance footage, has not been systematically evaluated. More experiments on diverse benchmarks are needed to establish broad applicability. Second, while our method achieves real-time inference on an NVIDIA RTX, practical UAV deployment often involves resource-constrained edge devices like Jetson TX2 or Xavier NX. The inference speed, latency, and memory consumption on such platforms have not been measured, so additional optimization or model compression may be required for onboard real-time processing. Third, our preliminary analysis shows that the model occasionally fails to detect extremely tiny objects (e.g., those smaller than 16×16 pixels) or heavily overlapping large objects. Extreme scale variations remain challenging for the current design, indicating that further improvements in multi-scale feature adaptation are needed. Addressing these limitations is the primary direction of our future research.

## 5. Conclusions

In this study, we present LHA-YOLO, a lightweight and accurate object detection framework built upon the YOLO11 baseline. The network is designed for UAV-based small-object detection, aiming to improve accuracy while maintaining real-time performance. Specifically, we propose the Lightweight Feature Extraction Module (LFEM), which uses a parallel spatial-channel attention mechanism within MDFR blocks to effectively capture discriminative features of small objects with low computational cost. We also introduce the Divide-and-Conquer Propagation Path (DCPP) strategy, which decouples semantic and spatial information flows through channel-guided and spatial-guided attention modules and a progressive aggregation scheme. This design resolves the conflict between semantic and spatial information in multi-scale feature fusion. We conduct extensive experiments on the VisDrone and UAVDT datasets and analyze the results both quantitatively and qualitatively. The experimental results confirm that LHA-YOLO outperforms the baseline YOLO11 models and many mainstream detectors. Compared to other advanced methods, LHA-YOLO achieves higher detection accuracy with competitive model complexity and inference speed.

Despite these satisfactory results, we find room for further improvement. The computational complexity of LHA-YOLO, especially from the DCPP attention modules, could be reduced. The model also occasionally fails on extremely small objects and densely overlapping scenes. In future work, we plan to incorporate more efficient attention designs or model pruning techniques to lower the computational overhead. We will also explore stronger multi-scale feature adaptation and loss functions to improve performance on dense- and tiny-object scenarios.

## Figures and Tables

**Table 1:** Performance comparison between LHA-YOLO network and YOLO11 network.

Methods	P (%)	R (%)	mAP50 (%)	mAP50–95 (%)	Params (M)	GFLOPs	FPS
YOLO11n	44.6	33.8	33.3	19.4	2.58	6.3	526
LHA-YOLOn	44.6	34.0	34.0	19.8	2.5	5.8	588
YOLO11s	50.8	38.6	39.4	23.5	9.42	21.3	500
LHA-YOLOs	52.7	39.6	41.6	24.9	9.0	18.8	556
YOLO11m	55.4	42.2	43.6	26.6	20.04	67.7	333
LHA-YOLOm	56.8	43.2	45.3	28.0	18.5	58.3	357
YOLO11l	56.3	44.1	45.3	27.9	25.3	86.6	204
LHA-YOLOl	57.6	44.1	46.0	28.2	21.7	68.4	286
YOLO11x	57.3	44.9	46.6	29	56.8	194.5	122
LHA-YOLOx	59.5	45.7	47.7	29.5	48.8	153.4	167

**Table 2:** Generalizability evaluation of the proposed LFEM and DCPP modules tested on YOLO8 and YOLO11.

Model	Baseline	LFEM	DCPP	mAP50 (%)	mAP50–95 (%)	Params (M)	GFLOPs
YOLO8s	✓	×	×	38.8	23.2	11.1	28.5
	✓	✓	×	39.3	23.6	7.6	18.2
	✓	×	✓	39.7	23.9	11.8	27.8
	✓	✓	✓	40.4	24.1	8.3	17.5
YOLO11s	✓	×	×	39.4	23.5	9.42	21.3
	✓	✓	×	39.8	23.8	8.58	19.4
	✓	×	✓	40.4	24.3	9.81	21.6
	✓	✓	✓	41.6	24.9	9.0	18.8

Note: ✓ denotes the adoption of the corresponding strategy, while × indicates its omission.

**Table 3:** Ablation study of MDFR block components across YOLO11 architectures on VisDrone dataset.

Model	PConv-PWConv	CA	SA	mAP50 (%)	mAP50–95 (%)	Params (M)	GFLOPs
YOLO11n	×	×	×	33.3	19.4	2.58	6.3
	✓	×	×	32.8	19.0	2.2	5.4
	✓	✓	×	33.6	19.5	2.4	5.9
	✓	×	✓	33.2	19.0	2.3	5.8
	✓	✓	✓	33.6	19.2	2.37	6.1
YOLO11s	×	×	×	39.4	23.5	9.42	21.3
	✓	×	×	39.1	23.3	7.8	17.7
	✓	✓	×	39.7	23.9	8.24	19.1
	✓	×	✓	39.6	23.5	8.1	19.1
	✓	✓	✓	39.8	23.8	8.58	19.4
YOLO11m	×	×	×	43.6	26.6	20.04	67.7
	✓	×	×	44.0	26.7	16.9	55.8
	✓	✓	×	44.6	27.2	17.6	58.6
	✓	×	✓	44.5	27.2	17.2	58.6
	✓	✓	✓	44.8	27.5	18	60

Note: ✓ denotes the adoption of the corresponding strategy, while × indicates its omission.

**Table 4:** Ablation study on CASA and SADA modules across YOLO11s on VisDrone dataset.

Model	Top-Down Pathway				Bottom-Up Pathway				mAP50 (%)	mAP50–95 (%)
	DPCA	DPSA	CMFF	PMFA	DPCA	DPSA	CMFF	PMFA		
YOLO11s	×	×	✓	×	×	×	✓	×	39.4	23.5
	✓	×	✓	×	×	×	✓	×	39.7	23.7
	✓	×	✓	×	×	✓	✓	×	39.9	24.0
	✓	×	×	✓	×	✓	×	✓	40.4	24.3
	✓	✓	×	✓	✓	✓	×	✓	40.1	23.9

Note: ✓ denotes the adoption of the corresponding strategy, while × indicates its omission.

**Table 5:** Comparison of our LHA-YOLO11 with other state-of-the-art object detectors on VisDrone dataset.

Model	mAP50 (%)	mAP50–95 (%)	Params (M)	GFLOPs
Faster RCNN [10]	19.6	-	41.2	118.8
Cascade RCNN [11]	18.9	-	69.0	146.6
YOLOv5s [29]	38.9	23.2	9.12	23.8
YOLOv6s [31]	37.0	22.0	16.3	43.7
YOLOv8s [16]	38.8	23.2	11.1	28.5
YOLOv9s [32]	40.4	24.1	7.17	26.7
YOLOv10s [33]	38.8	23.4	7.22	21.4
YOLOv11s [24]	39.4	23.5	9.42	21.3
TPH-YOLOv5s [63]	39.3	23.6	9.2	23.1
FFCA-YOLOs [64]	37	-	2.33	17.4
PS-YOLOs [42]	40.7	24.2	5.53	20.0
RT-DETR-R18 [52]	41.4	23.2	19.9	57.0
LHA-YOLOs	41.6	24.9	9.0	18.8

**Table 6:** Comparison of our LHA-YOLO11 with other state-of-the-art object detectors on UAVDT dataset.

Model	mAP50 (%)	mAP50–95 (%)	Params (M)	GFLOPs
YOLOv5s [29]	31.3	17.7	9.12	23.8
YOLOv6s [31]	30.8	17.8	16.3	43.7
YOLOv8s [16]	30.6	18.0	11.1	28.5
YOLOv9s [32]	29.6	17.6	7.17	26.7
YOLOv10s [33]	31.6	19.3	7.22	21.4
YOLOv11s [24]	32.2	19.4	9.42	21.3
RT-DETR-R18 [52]	30.3	17.6	19.9	57.0
LHA-YOLOs	36.9	22.9	9.0	18.8

## Data Availability

Data are contained within the article.

## Abbreviations

The following abbreviations are used in this manuscript:

UAV	Unmanned aerial vehicle
LHA-YOLO	Lightweight and high-accuracy network based on YOLO11
LFEM	Lightweight feature extraction module
DCPP	Divide-and-conquer propagation path
CASA	Channel attention-guided semantic aggregation
SADA	Spatial attention-guided detail aggregation
MDFR	Multi-dimension feature representation
DPCA	Dual-pooling channel attention
DPSA	Dual-pooling spatial attention
PConv	Partial convolution
PWConv	Pointwise convolution
mAP	Mean average precision
GFLOPs	Gigabit floating point operations
